# Impacts of Electroextraction Using the Pulsed Electric Field on Properties of Rice Bran Protein

**DOI:** 10.3390/foods12040835

**Published:** 2023-02-15

**Authors:** Saban Thongkong, Wannaporn Klangpetch, Kridsada Unban, Pipat Tangjaidee, Yuthana Phimolsiripol, Pornchai Rachtanapun, Kittisak Jantanasakulwong, Regine Schönlechner, Parichat Thipchai, Suphat Phongthai

**Affiliations:** 1 Faculty of Agro-Industry, Chiang Mai University, Chiang Mai 50100, Thailand; 2 The Cluster of Agro Bio-Circular-Green Industry (Agro BCG), Chiang Mai University, Chiang Mai 50100, Thailand; 3 Institute of Food Technology, Department of Food Science and Technology, University of Natural Resources and Life Sciences, 1190 Vienna, Austria; 4 Doctor of Philosophy Program in Nanoscience and Nanotechnology (International Program/Interdisciplinary), Faculty of Science, Chiang Mai University, Chiang Mai 50200, Thailand; 5 Lanna Rice Research Center, Chiang Mai University, Chiang Mai 50100, Thailand

**Keywords:** rice protein, pulsed electric field, protein hydrolysates, antioxidant peptide, in vitro GI digestion

## Abstract

The pulsed electric field (PEF) was applied to improve the extraction yield and properties of rice bran proteins from two rice varieties (“Kum Chao Mor Chor 107” and “Kum Doi Saket”). As compared to the conventional alkaline extraction, PEF treatment at 2.3 kV for 25 min increased the protein extraction efficiency by 20.71–22.8% (*p* < 0.05). The molecular weight distribution detected by SDS-PAGE and amino acid profiles of extracted rice bran proteins was likely unchanged. The PEF treatment influenced changes in the secondary structures of rice bran proteins, especially from the β-turn to the β-sheet structure. Functional properties of rice bran protein including oil holding capacity and emulsifying properties were significantly improved by PEF treatments by about 20.29–22.64% and 3.3–12.0% (*p* < 0.05), respectively. Foaming ability and foam stability increased by 1.8- to 2.9-fold. Moreover, the in vitro digestibility of protein was also enhanced, which was consistent with the increment of DPPH and ABTS radical-scavenging activities of peptides generated under in vitro gastrointestinal digestion (37.84–40.45% and 28.46–37.86%, respectively). In conclusion, the PEF process could be a novel technique for assisting the extraction and modification of the protein’s digestibility and functional properties.

## 1. Introduction

Rice (*Oryza sativa* L.) is an important crop for humans, particularly in Asian countries. Fundamentally, 68–70% of white rice is obtained from a milling process of paddy rice; meanwhile, rice husks and rice bran are classified as low-value byproducts. These byproducts have long been recognized as unsuitable for human consumption but are used in animal feed or biofertilizers. However, several studies have reported the valorization of rice bran as a raw material for some high-value ingredients, such as oil, α-tocopherol, γ-oryzanol, and phenolics [1,2,3]. These compounds showed high protective effects against cancer formation and coronary heart disease [2]. As over 50 million tons of rice bran is produced each year, the rice bran oil industry is rapidly expanding. Solvent extraction and compression have been popularly used as methods for extracting rice bran oil. However, rice bran residue still contains a variety of beneficial nutrients such as proteins, polysaccharides, vitamins, minerals, etc. [3,4]. High-quality proteins in rice bran, with a high protein efficiency ratio (PER) of 1.6–1.9 compared to casein, are reported in a range of 12 to 15%. Moreover, the capability of rice bran protein to resist oxidation or even inhibit the formation of cancer cells has been reported [3,5]. Therefore, the valorization and recovery of protein from defatted rice bran is an attractive area that is currently being studied in rice-consuming countries.

Generally, protein extraction is conducted using conventional alkaline extraction; unfortunately, this technique provides a low extraction yield. Thus, novel techniques, such as microwave, ultrasonic, and supercritical fluid extraction, have been used in the extraction of rice bran protein [6,7,8]. However, some methods have several drawbacks, including the cost of operation, disturbance sound, and heat generation during the extraction process that can cause losses of protein quality and functional properties. Enzymatic hydrolysis has been used for the modification of the functional properties of protein. On the basis of our previous studies [7,9], partial enzymatic hydrolysis can be advantageous for the production of a protein with desired functional properties such as foaming ability and emulsifying properties. Unfortunately, there are also disadvantages, e.g., longer hydrolysis time, enzymes that are more expensive and very sensitive, and the formation of some bitter byproducts. Therefore, a possible process that can overcome these challenges is required for the modification of protein functional properties.

Pulsed electric field (PEF) technology is a non-thermal process based on a short pulsing of high electric field strength delivered to the targets of plant materials placed between two pieces of electrodes conducted at room temperature. The intensity of the electric field generated between the electrodes affects the transmembrane potential differential exceeding a critical value occurring in pore formation called “electroporation” on the cell membrane in a short time, and this process can be reversible or irreversible [10]. According to this principle, the PEF mechanism has a high potential to be used for microbial inactivation in liquid food, the improvement of raw material structures for the short-time drying process, a reduction in oil absorption in fried food, the enhancement of fermentation speed, the peeling of fruits and vegetables, and a reduction in some pesticides, e.g., methamidophos (9.1%) and chlorpyrifos (9.0%) in apple juice [11,12]. In addition, it has been applied to improve the bioactive compound extraction from plant material [13]. The use of PEF to improve the physicochemical and functional properties, including solubility, oil-holding capacity, emulsion stability, foamability, and water-holding capacity, of proteins from canola, whey protein, wheat gluten, and crickets has been previously addressed [14,15,16]. However, the application of PEF for rice bran protein extraction and its effects on protein properties has not yet been studied. Therefore, this study aims to assess the potential of a pulsed electric field to improve rice bran protein extraction yield as well as to investigate its effect on physicochemical, functional properties, and digestibility under in vitro simulated gastrointestinal digestion of rice bran protein.

## 2. Materials and Methods

### 2.1. Materials

Two varieties of rice bran, including “Kum Chao Mor Chor 107” (16.69 ± 0.17% protein, 13.66 ± 0.00% fat, 10.28 ± 0.22% crude fiber, 10.04 ± 0.06% ash) and “Kum Doi Saket” (18.05 ± 0.28% protein, 11.69 ± 0.06% fat, 8.83 ± 0.13% crude fiber, 9.90 ± 0.11% ash), were obtained from the Lanna Rice Research Center, Chiang Mai University, Thailand. Trypsin (E.C.3.4.21.4, ≥10,000 BAEE units/mg protein) from bovine pancreas, pepsin (E.C.3.4.23.1, ≥250 units per milligram solid) from porcine gastric mucosa, and 2,20-azino-bis (3-ethylbenzthiazoline-6-sulphonic acid), or ABTS, and 1,1-diphenyl-2-picrylhydrazyl, or DPPH, were purchased from the Sigma-Aldrich Company (St. Louis, MO, USA).

### 2.2. Rice Bran Preparation

Rice bran was dried in a hot air oven at 60 °C for 8 h and defatted with 95% ethanol (1:5, *w*/*v*) with an overhead stirrer for 1 h. The slurry was centrifuged at 6000 rpm for 15 min. The residue was collected and dried in a hot air oven for 5–6 h. The resulting defatted rice bran was stored in a plastic zip-lock bag at −18 °C before use in further experiments.

### 2.3. Electroextraction of Rice Bran Protein Using Pulsed Electric Field (PEF)

Rice bran was dispersed in distilled water (1:10 *w*/*v*), and the pH was adjusted to 10 using 1 M NaOH. The mixtures (150 mL) were transferred into different sizes of acrylic-treatment chambers equipped with two stainless steel electrode plates (3.47 and 6.15 cm distance between electrodes). A bench-scale pulsed electric field system (TSUS Febix Foodtech Co., Ltd., Chiang Mai, Thailand) was operated at constant pulses and an operating voltage at 250 pulses/min and 8 kV, respectively, achieving the electric fields of 1.3 kV/cm and 2.3 kV/cm. The treatment times were varied at 15, 20, and 25 min, thus avoiding the effect of heat generated during PEF treatments on protein properties; afterward, the mixture was further continuously stirred until 1 h had passed. The derived mixture was centrifuged at 6000 rpm for 15 min, and the supernatant was collected before the pH was adjusted to 4.5 with 1.0 M hydrochloric acid and was centrifuged suddenly. The precipitated protein was neutralized and dried in a freeze dryer, and the resulting rice bran protein was stored at −18 °C in a plastic zip-lock bag.

The alkaline extraction condition reported by Phongthai et al. [7] was used as a comparative method. Rice bran was mixed with distilled water (1:10, *w*/*v*), and the pH was adjusted to 10 using sodium hydroxide. The suspension was stirred using a magnetic stirrer at room temperature for 1 h and then centrifuged at 6000 rpm for 15 min. The supernatant was collected and adjusted to attain a pH of 4.5 using hydrochloric acid, and the supernatant was then centrifuged under the same conditions. The precipitate was adjusted to neutral pH before being freeze-dried. The dried powder was stored at room temperature in a plastic zip-lock bag. The percentages of protein increments were calculated by using the following equation:

(1) $$\mathrm{Protein}\;\mathrm{increment}\;\left(\%\right)=\frac{(\mathrm{protein}\;\mathrm{in}\;\mathrm{PEF}\;\mathrm{treatments}\;-\;\mathrm{protein}\;\mathrm{in}\;\mathrm{ALK}\;\mathrm{treatments})\times 100}{\left(\mathrm{protein}\;\mathrm{in}\;\mathrm{ALK}\;\mathrm{treatments}\right)}$$

### 2.4. Rice Bran Morphology Analysis

The micrographs of PEF-treated and untreated rice bran samples were taken using a scanning electron microscope (Inspect™ S50, FEI Company, Hillsboro, OR, USA) at an accelerating voltage of 15 kV with a magnification of ×1000 to observe the effect of PEF on rice bran structure.

### 2.5. Protein Pattern Using SDS-PAGE

The patterns of proteins in ALK-treated and PEF-treated solutions were investigated using sodium dodecyl sulfate-polyacrylamide gel electrophoresis (SDS-PAGE) as described by Laemmli [17]. The protein solutions were mixed with a sample buffer (0.125 M Tris-HCl, pH 6.8, 4% SDS, and 20% glycerol) and loaded onto 12% separation gel and 4% stacking gel. The electrophoresis was run under a constant current of 15 mA per gel. Coomassie Brilliant Blue R-250 solution and acetic acid/methanol solution were used as staining and de-staining solutions, respectively. To ascertain the molecular weight of protein, the electrophoretic mobility of protein samples was compared with known molecular weight protein.

### 2.6. Amino Acid Composition Analysis

In total, 17 amino acids in ALK-treated and PEF-treated samples were separated on a Zebron ZB-AAA GC column (10 mm × 0.25 mm, 0.25 µm film thickness) and analyzed by GC–MS (6890N; Agilent Technologies, Santa Clara, CA, USA).

### 2.7. Functional Properties Determinations

Functional properties of ALK-treated and PEF-treated proteins were determined following the method of Phongthai et al. [9] with some modifications.

#### 2.7.1. Oil Holding Capacity

Protein samples (0.5 g) were mixed with 10 mL of soybean oil. The mixtures were centrifuged at 2000 rpm for 15 min. The unbound oil was decanted slowly. Oil holding capacity was calculated in terms of the ratio of absorbed oil and protein powder used (*g*/*g*).

#### 2.7.2. Foaming Properties

Protein sample solutions (1% *w*/*v*, 20 mL) were homogenized in a 60 mL beaker at 10,000 rpm for 1 min and then transferred to a 100-mL cylinder. The total volume was measured at 0 and 30 min after whipping. The foaming activity and foam stability were calculated using the following equations:

Foaming activity = [(A − B)/B] × 100(2)

Foam stability = [(A_30min_ − B)/(A_0min_ − B)] × 100 (3)

where A is the volume after whipping (mL), and B is the volume before whipping (mL).

#### 2.7.3. Emulsifying Properties

Protein solutions (1% *w*/*v*, 10 mL) were mixed with 10 mL soybean oil and then homogenized at 10,000 rpm for 1 min. The emulsifying properties were calculated using the following equations:

Emulsifying activity = (A/B) × 100(4)

Emulsifying stability = [(A_incubate_/A)] × 100 (5)

where A is the volume of emulsified layer, A_incubate_ is the volume of the emulsified layer after incubation at 80 °C for 10 min, and B is the total volume.

### 2.8. Secondary Structure Changes Using Fourier-Transform Infrared Spectroscopy (FTIR)

The transmission infrared spectra of the protein samples were measured using an FTIR spectrometer (Tensor 27, Bruker, Ettlingen, Germany). The samples (~2 mg) were mixed with KBr and pressed into a pellet. The measurement was performed in the range of 400–4000 cm^−1^, with a resolution of 4 cm^−1^. The software OriginPro 2022 (OriginLab Corporation, Northampton, MA, USA) was used to separate the spectra in the amide I region, between 1600 and 1700 cm^−1^, into multi-component peaks according to a specific wave number as follows: α-helices at 1657 cm^−1^, β-sheets at 1611 and 1626 cm^−1^, β-turns at 1673 and 1688 cm^−1^, and random coils at 1642 cm^−1^, as described in Qian et al. [18].

### 2.9. In Vitro Gastrointestinal Digestion

The digestion of proteins was based on the method of Xia et al. [19]. The pH of 1% protein solutions was adjusted to 1.5 using 3.0 M HCl. Pepsin was added to the pre-incubated protein solutions (enzyme: protein ratio of 1:100, *w*/*w*) at 37 ± 2 °C. The digestion time was controlled for 120 min. The mixture was then neutralized with 3.0 M NaOH; afterward, trypsin was added to the previously digested mixture (enzyme: protein ratio of 1:100, *w*/*w*). The second digestion was conducted at 37 ± 2 °C for 120 min, and then the trypsin activity was terminated with heating at 95 ± 1 °C for 10 min. The digested protein solution was dried using a freeze dryer, and the resulting protein powder was stored at −18 °C in a plastic zip-lock bag.

### 2.10. Antioxidant Activity Determinations

The DPPH radical-scavenging activity was assessed following the method of Phongthai et al. [9]. The digested and non-digested proteins (1 mg/mL, 0.5 mL) were mixed with 0.1 mM DPPH (2 mL) and then incubated for 30 min (dark condition). Distilled water was used as a control. The absorbance was read at 517 nm (Genesys™ 10S, Thermo Scientific, Waltham, MA, USA). The percentage of inhibition activity was calculated using the following equation:

Inhibition activity (%) = [(Abs_control_ − Abs_sample_)/(Abs_control_)] × 100 (6)

where Abs_control_ and Abs_sample_ are the absorbances of the control and sample, respectively.

The ABTS radical-scavenging activity was assessed as described in Phongthai et al. [9]. Potassium persulfate (2.6 mM) and 7.4 mM ABTS solutions were mixed (1:1) and incubated for 12 h. The working solution was prepared by diluting the stock solution in DI water at a ratio of 1:60. The digested and non-digested protein solution (150 µL) was mixed with 28.5 mL of the working solution and incubated for 2 h in the dark. The absorbance was measured at 734 nm (Genesys™ 10S, Thermo Scientific, Waltham, MA, USA). The percentage of inhibition activity was calculated using the following equation:

Inhibition activity (%) = [(Abs_control_ − Abs_sample_)/(Abs_control_)] × 100 (7)

where Abs_control_ and Abs_sample_ are the absorbances of the control and sample, respectively.

### 2.11. Statistical Analysis

The PEF and ALK extraction were conducted in duplicate. The characterizations of the derived protein fractions were performed in triplicate (n = 6). Statistical analysis was performed using analysis of variance (ANOVA). Duncan’s multiple range test (DMRT) was used to compare the means at a 95% confidence level (SPSS Version 17.0).

## 3. Results and Discussion

### 3.1. Effect of PEF Treatment on Protein Extraction

The amounts of protein extracted by ALK and different PEF conditions are reported in Table 1.

**Figure 1:**
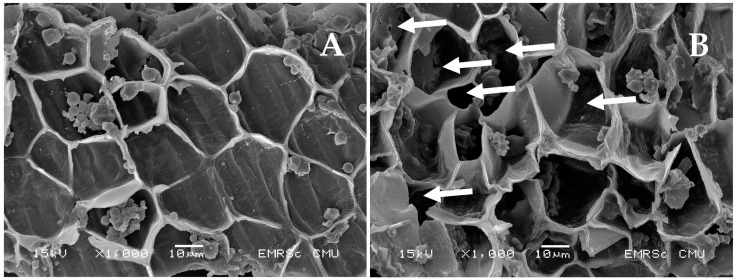
Rice bran characteristics treated by ALK (**A**) and PEF (**B**) treatments under SEM (×1000).

It was found that electroextraction using PEF treatments significantly increased the protein extraction yield (*p* < 0.05), especially when compared to that of ALK. The protein extractability using PEF tended to increase as the treatment time was longer extended. However, the maximum PEF-treated time was limited to 25 min to prevent heat generation that could cause unwanted denaturation of a native protein. Moreover, the number of extracted proteins increased as the electric field strength increased. The highest protein yields of both varieties of rice (2485.82–2980.73 mg) were obtained from the following conditions: 2.3 kV/cm and 25 min of treatment time (*p* < 0.05). PEF has been used to improve protein extraction efficiency from many sources. Psarianos et al. [20] reported that the application of PEF treatment at 1.5 kV/cm increased the extraction yield from cricket (*Acheta domesticus*) by about >18% after 60 min of extraction. This is due to the electroporation phenomenon that occurs when the plant cells are exposed to an electric field that induces an electric potential. When the potential reaches a critical value, pores are formed in the weak areas of the membrane [21]. The smaller size of the pore may appear from using a low-intensity PEF treatment, whereas larger pores can be formed upon increasing the strength of the electric field and treatment time [22]. According to a microstructure image taken by SEM (Figure 1), it was found that the ALK-treated rice bran cell wall had an integral block structure (2A); meanwhile, the appearance of the exposed pores surrounding the rice bran cell wall was clearly observed for the PEF-treated sample (2B), as indicated by a white arrow. The disruption of Spirulina’s cell integrity by PEF was also found clearly under SEM [21]. These opened structures might be a major cause of the acceleration of the protein solubilization and release in comparison to the PEF-untreated sample (ALK) by about 7.87 to 22.80%. However, Lam et al. [23] reported that PEF treatment at 7.5–30 kV/cm enhanced protein release from microalgae *Chlorella vulgaris* only up to 13%. This is because there are various factors besides processing parameters that affect the extraction yield, particularly raw material properties, such as the number and thickness of cell walls, even in the same plants, that may be varied in each growth phase [24], and the types of cell membranes or walls [22]. Therefore, PEF treatment at 2.3 kV/cm and a treatment time of 25 min was chosen to produce the rice bran protein for further investigation of its effect on protein properties.

### 3.2. Effect of PEF on Amino Acid Composition

Amino acid profiles of two-variety rice bran proteins derived from ALK and PEF treatments are shown in Table 2. It was found that the amino acid contents of each rice variety between the ALK and PEF treatments were likely similar. The PEF treatment did not change the ratio of essential amino acids to non-essential amino acids, which was 0.62 for “Kum Chao Mor Chor 107” and 0.55 for “Kum Doi Saket”. Moreover, the percentages of essential amino acids in proteins were found to be constant at 38 and 35, respectively. Therefore, it can be concluded that PEF treatment at an electric field strength between 1.3 and 2.3 kV/cm did not affect the amino acid compositions of the derived rice bran proteins. This result is in agreement with previous studies [25,26,27] that PEF treatments at 5 kV and 10 kV showed no significant change in the amino acid compositions of meats from dairy cows and red deer.

Moreover, the amino acid profiles of these two-variety rice bran proteins were very similar, except for the proline content. As a result, the proline content in the “Kum Doi Saket” variety’s protein was 2.74 to 3.95-fold higher than that of “Kum Chao Mor Chor 107” (*p* < 0.05). In addition, the results revealed that leucine, valine, and lysine content were found to be major essential amino acids for both rice varieties.

### 3.3. Effect of PEF on Protein Pattern

**Figure 2:**
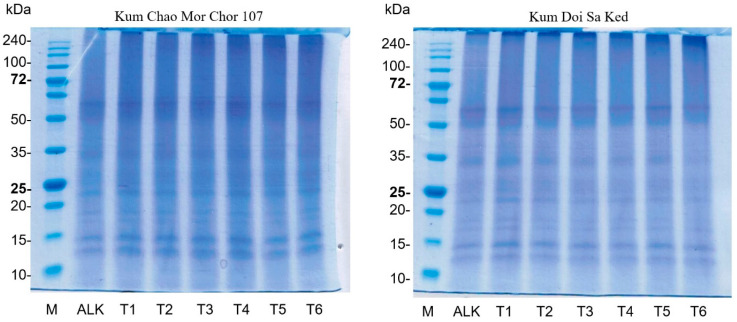
Patterns of rice bran proteins derived from ALK and different PEF treatments (T1: 2.3 kV/cm + 15 min; T2: 2.3 kV/cm + 20 min; T3: 2.3 kV/cm + 25 min; T4: 1.3 kV/cm + 15 min; T5: 1.3 kV/cm + 20 min; T6: 1.3 kV/cm + 25 min).

SDS-PAGE, under non-reducing conditions, was used to investigate the pattern of rice bran proteins (Figure 2). The protein patterns of both varieties were similar. The major bands were found at between 10 and 15 kDa and 62 kDa. Similarly, the patterns showed no obvious changes in molecular weight distribution among protein samples obtained from ALK and PEF treatments at 1.3–2.3 kV/cm. This result is consistent with the finding of Wu et al. [28] that no new bands of egg white protein appeared after treatment at an electric field strength of 25 kV for 400–600 µs. Dong et al. [29] observed no significant band change in the myofibrillar protein from chicken meat treated with and without PEF at 8–28 kV/cm. Furthermore, the application of PEF treatment at 35 kV did not even change the protein profiles of the whey protein isolate [14]. However, these results differ from those from a study of Zhang et al. [15], as a new band of canola protein appeared after PEF treatment. This is probably due to the aggregation of protein caused by the long treatment time at a high electric field strength.

### 3.4. Effect of PEF on Secondary Structure

**Figure 3:**
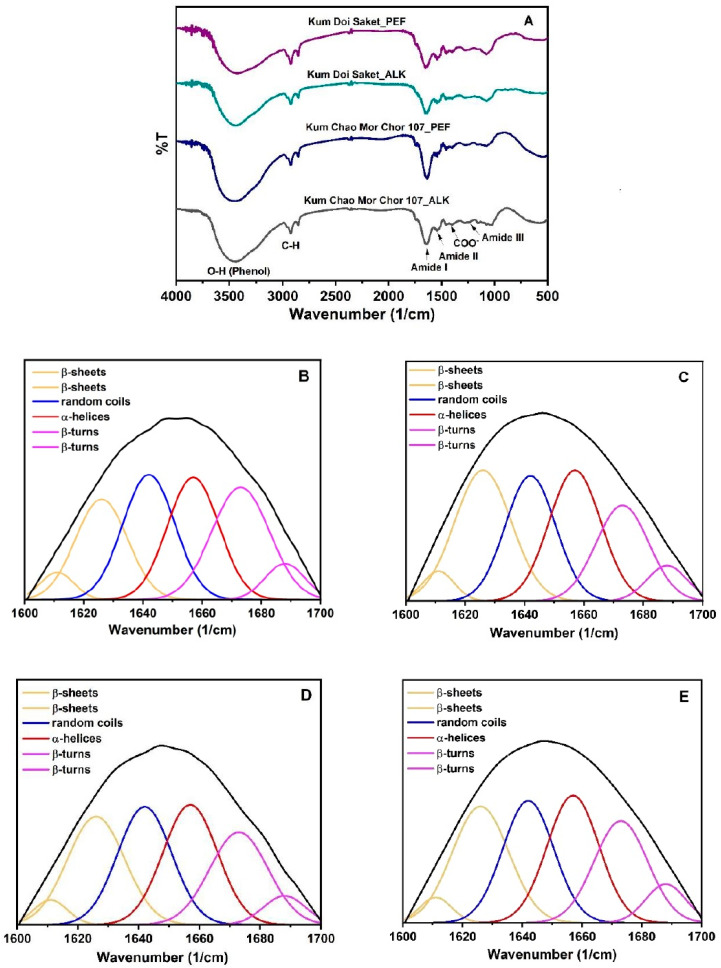
FTIR spectra (**A**) and peak-fitting of the secondary structure curves of “Kum Chao Mor Chor 107” protein from ALK (**B**) and PEF (**C**) treatments, and “Kum Doi Saket” Protein from ALK (**D**) and PEF (**E**) treatments.

The FTIR spectra of ALK- and PEF-treated protein samples are shown in Figure 3A. The baseline adjusting, deconvolving, and second-derivative fitting were conducted using OriginPro 2022 software. The Gaussian model was chosen for multi-component division. Six peaks of four components of rice bran proteins were derived, as illustrated in Figure 3B–E. The peak areas were used to calculate the portion percentage of each component including α-helices, β-sheets, β-turns, and random coils, as summarized in Table 3.

As a result, the secondary structures of rice bran proteins were influenced by the PEF treatment. The α-helices protein slightly increased, whereas the portion of β-sheets significantly increased in PEF-treated proteins (*p* < 0.05). This result is related to the results from a study by Qian et al. [18]: the α-helices and β-sheets in egg white protein slightly increased at a low voltage (5 kV). However, the α-helices tended to decrease at an applied higher voltage (10–25 kV), but the β-sheets continuously increased by 43.1%. Liang et al. [30] reported that the contents of α-helices and β-sheets in pine nut protein, which were detected by circular dichroism spectroscopy, also increased following the PEF treatments. On the other hand, the reductions in β-turns and random coils structures in chicken breast meat after PEF treatment were reported by Dong et al. [29], which is consistent with the findings of this study. These two portions in rice bran proteins of both varieties were significantly reduced by the PEF treatments (*p* < 0.05), suggesting that the PEF treatment caused the secondary structural transformations of proteins, especially from β-turns to β-sheets. This may be due to the destabilization of the hydrogen bond between the N-H and C=O of residues i and i + 3 found in the β-turn structure via the electroporation effect. However, the PEF treatment may differently alter the secondary structure of the proteins according to the PEF processing parameters used, amino acid composition, and inter- and intra-molecular interactions in protein structures. Therefore, the characterization by determining reactive sulfhydryl content and surface hydrophobicity of the treated and untreated proteins might be another insight explaining protein denaturation based on the changes in their tertiary structure.

### 3.5. Effect of PEF on Functional Properties

The effects of PEF on the functional properties of rice bran proteins are summarized in Table 3. PEF treatments unambiguously and significantly affected the oil holding capacity (*p* < 0.05). As compared to that of ALK, the oil-holding capacities of “Kum Chao Mor Chor 107” and “Kum Doi Saket” proteins were significantly improved by 22.64% and 20.29%, respectively (*p* < 0.05). This impact has also been mentioned in several previous studies. Zhang et al. [15] reported that the oil-holding capacity of canola proteins treated with PEF at 25 kV was increased up to 4.92–6.03 mL/g oil in each sample and remained steady after further increased electric field strength. Moreover, the application of PEF at 2.5–12.5 kV, pulse width at 2–10 µs, and 1–9 min of residence time significantly enhanced the capacity of gluten proteins to hold oil in their structures [16]. A similar trend was reported for cricket flour (72.45% protein), in that the samples treated with PEF treatments at 1.5 kV/cm increased the oil holding capacity from 2.27 to 3.21 g of oil per gram of sample, which is a 41.3% increase [20].

In addition, PEF treatment at 2.3 kV/cm for 25 min in this study showed a significant positive effect on the foam formation and stabilization of these two proteins (*p* < 0.05), as it was increased by 2.48- to 2.85-fold and 1.79- to 1.83-fold, respectively. This result is in line with the secondary structure change from random coils to α-helices and β-sheets (Section 3.4). This unfolded state is important for the elasticity of the protein network structure [19] that could support the air-enveloping and stabilizing foam structure. For consistency, Zhang et al. [15] indicated that the foaming capacity and foam stability of canola protein increased with the voltage and resident time of 30 kV and 180 s. Zhang et al. [16] revealed a significant improvement in foaming capacity and foam stability of wheat gluten protein using electric field intensities of 7.5–12.5 kV, pulse frequencies of 300–900 Hz, and pulse widths of 2–10 µs.

Furthermore, it was observed that the ability of rice bran proteins to emulsify the immiscible liquid of water and oil, as well as to stabilize the emulsion systems was significantly improved by PEF treatment (*p* < 0.05). The emulsifying ability was 3.3–5.3% improved, reaching the maximum value of 100%; meanwhile, emulsion stability was improved by about 9.6–12.0%. Similarly, canola and gluten proteins treated with 10 kV of electric intensity showed better functionality for emulsion properties [15,16]. Psarianos et al. [20] reported that a different range of energy input during PEF treatment could increase the emulsion capacity of high-protein cricket flour by 22.1–74.7%. However, no difference was found in whey protein isolate treated with and without PEF processing parameters, including 30 kV/cm and 19.2–211 µs [14]. This may be attributed to the lower hydrophobic–hydrophilic balance of amino acids in whey protein (0.41), as reported by Chungchunlam et al. [31], when compared to the proteins used in this study (0.43–0.45).

As a result, these improvements are probably due to the structural changes of protein, e.g., the buried SH group’s exposure and partial unfolding that increase surface hydrophobicity [32]. The unfolding of protein molecules caused by PEF creates hydrophilic or hydrophobic inner groups, which are beneficial for the formation of an interface between air and water. The interactions of the extending molecules construct a steady two-dimensional network and interfacial membranes, which directly affect protein functional properties, including oil-holding capacity, emulsifying, and foaming properties [16]. According to the previously reported studies regarding the effect of various PEF processing parameters on protein properties, it can be summarized that the voltage and residence time are two variables that observably improved the protein functionality, whereas the pulse frequency and width affect the functional properties of the protein in a smaller degree. However, in essence, the specific composition and the hydrophobic–hydrophilic balance of amino acids are the main factors for predominant expressing protein functional properties.

### 3.6. In Vitro Digestibility and Antioxidant Properties

According to our previous study, the in vitro digestibility of rice bran protein was analyzed and reported to be about 60.92–62.51% [7]. In this study, the result revealed the impact of PEF treatment on the enhancement of both rice bran proteins’ digestibility and their antioxidant properties. The increments of inhibition activity on the DPPH and ABTS radicals of the digested proteins and non-digested proteins were calculated and are presented in Table 3. Digested PEF-derived proteins showed a significantly higher ability to scavenge those free radicals than that of ALK (*p* < 0.05). This is because the PEF treatment induced protein structural changes, especially unfolding, which influences the protease active sites [26], resulting in more susceptibility to simulated in vitro gastrointestinal digestion, then allowing the release of potent antioxidant peptides. Therefore, the increment of antioxidant activities directly reflects the digestibility improvement of PEF-treated proteins. Similar findings were found in whey protein isolate, beef, and red deer muscles [14,25,26,27]. However, a massive protein unfolding and greater aggregation can cause a reduction in proteolytic cleavage [25]. Apart from the PEF treatment, the amino acid composition of different proteins is likewise a critical parameter to determine digestibility, as trypsin mainly cleaves peptide bonds at the carboxyl side of lysine and arginine, whereas pepsin digests the bonds between hydrophobic and aromatic amino acids such as tyrosine, tryptophan, and phenylalanine. Therefore, the improvement in digestibility using PEF treatment may not be reached for proteins without these specific amino acids in their sequences.

## 4. Conclusions

Converting protein-containing agricultural byproducts to an alternative protein source has recently been a challenging food sustainability goal. Modification of protein structures using a new technique for desired functional properties has also gained attention from the food ingredient industry. In this study, the recovery of rice bran protein through electroextraction using a pulsed electric field was successfully conducted. As compared to a conventional alkaline extraction, the PEF treatment at 2.3 kV for 25 min increased the protein extraction efficiency by 20.71–22.8%. In addition, the functional properties including oil holding capacity, emulsification, and foaming properties of the derived PEF-treated proteins were improved without affecting the nutritive value. The increments of antioxidant properties of PEF-treated proteins after in vitro gastrointestinal digestion proved the protein digestibility enhancement. It can be concluded that the pulsed electric field has a high potential for use as a novel process for extraction purposes, as well as a modification method of food protein functionality.

## Figures and Tables

**Table 1:** Extracted protein derived from ALK and PEF treatments.

Treatments	Treatment Time (min) PEF/ALK	Extracted Protein (mg)		Protein Increment (%)	
		Kum Chao Mor Chor 107	Kum Doi Saket	Kum Chao Mor Chor 107	Kum Doi Saket
ALK	0/60	2060.28 ± 80.94 ^D^	2427.51 ± 16.52 ^e^	-	-
	15/45	2281.70 ± 50.10 ^C^	2618.28 ± 24.78 ^d^	10.78 ± 1.92 ^D^	7.87 ± 1.75 ^c^
1.3 kV/cm	20/40	2323.50 ± 68.74 ^B,C^	2775.66 ± 57.23 ^c^	12.80 ± 1.09 ^C^	14.35 ± 2.49 ^a,b^
	25/35	2407.13 ± 65.60 ^A,B^	2875.81 ± 75.71 ^b,c^	16.86 ± 1.41 ^B^	18.47 ± 2.97 ^a^
	15/45	2289.20 ± 40.47 ^C^	2687.35 ± 50.73 ^c,d^	11.16 ± 2.40 ^C,D^	10.70 ± 1.58 ^b^
2.3 kV/cm	20/40	2367.89 ± 50.58 ^B,C^	2691.43 ± 14.58 ^c^	14.97 ± 2.06 ^C^	10.87 ± 0.87 ^b^
	25/35	2485.82 ± 35.25 ^A^	2980.73 ± 29.78 ^a^	20.71 ± 3.03 ^A^	22.80 ± 2.04 ^a^

Different letters in each column represent significantly different mean values (*p* < 0.05).

**Table 2:** Amino acid profiles of extracted rice bran proteins (mg/100 g sample).

Amino Acid	Kum Chao Mor Chor 107		Kum Doi Sa Ket	
	ALK	PEF	ALK	PEF
***Essential amino acids (EAA)***				
Histidine	640.20 ± 48.63	684.25 ± 109.96	722.04 ± 8.75	736.90 ± 13.93
Isoleucine	634.17 ± 77.90	672.08 ± 130.79	660.82 ± 6.23	739.26 ± 7.14
Leucine	1658.21 ± 252.24	1607.07 ± 306.33	1630.98 ± 27.83	1762.62 ± 12.49
Lysine	1073.06 ± 215.67	1028.56 ± 191.76	1127.11 ± 21.00	1112.95 ± 20.48
Methionine	287.60 ± 9.09	314.78 ± 74.47	342.34 ± 1.81	358.22 ± 2.23
Phenylalanine	998.18 ± 92.44	958.00 ± 186.67	958.68 ± 8.76	1058.55 ± 7.19
Threonine	856.49 ± 174.24	672.21 ± 103.87	751.54 ± 64.08	715.86 ± 5.02
Valine	1356.95 ± 21.41	1353.54 ± 273.08	1324.88 ± 2.33	1516.78 ± 3.34
**Sum**	**7504.86**	**7290.49**	**7518.39**	**8001.14**
***Non-essential amino acids*** ***(Non-EEA)***				
Aspartic acid	2022.32 ± 417.52	1762.14 ± 331.80	1846.45 ± 71.62	1936.99 ± 18.28
Serine	1059.88 ± 141.71	995.36 ± 194.39	1023.90 ± 23.69	1096.07 ± 14.58
Glutamic acid	3017.71 ± 157.71	3180.24 ± 609.82	3299.06 ± 4.39	3522.90 ± 25.40
Proline	722.95 ± 180.74	488.39 ± 65.75	1978.46 ± 116.23	1931.47 ± 3.76
Glycine	1228.99 ± 107.95	1267.13 ± 242.40	1327.04 ± 3.32	1403.36 ± 8.17
Alanine	1501.72 ± 222.36	1412.67 ± 279.68	1427.34 ± 8.17	1564.39 ± 15.99
L-Cystine	84.11 ± 36.20	62.98 ± 9.75	82.95 ± 10.18	68.06 ± 1.98
Tyrosine	579.68 ± 165.46	559.94 ± 92.45	592.01 ± 26.43	597.44 ± 4.19
Arginine	1869.05 ± 32.71	2016.62 ± 402.38	2127.96 ± 5.39	2250.20 ± 3.32
**Sum**	**12,086.41**	**11,745.47**	**13,705.17**	**14,370.88**
EAA:Non-EAA ratio	0.62 ± 0.01 ^ns^	0.62 ± 0.01 ^ns^	0.55 ± 0.08 ^ns^	0.55 ± 0.06 ^ns^
% Essential amino acid	38.31 ± 0.07 ^ns^	38.29 ± 0.08 ^ns^	35.42 ± 0.40 ^ns^	35.76 ± 0.02 ^ns^
**Total amino acid contents**	**19,591.27**	**19,035.96**	**21,223.56**	**22,372.72**

ns represents no significant difference.

**Table 3:** Secondary structure changes and functional and antioxidant properties of rice bran proteins.

Properties	Kum Chao Mor Chor 107		Kum Doi Sa Ket	
	ALK	PEF	ALK	PEF
***Secondary Structures***				
α-helices (portion, %)	23.47 ± 0.06 ^B^	24.37 ± 0.06 ^A^	24.29 ± 0.06 ^b^	24.64 ± 0.06 ^a^
β-sheets (portion, %)	22.84 ± 0.05 ^B^	29.94 ± 0.06 ^A^	24.87 ± 0.06 ^b^	27.13 ± 0.05 ^a^
β-turns (portion, %)	29.64 ± 0.07 ^A^	23.15 ± 0.06 ^B^	26.48 ± 0.06 ^a^	25.63 ± 0.06 ^b^
Random coils (portion, %)	24.23 ± 0.06 ^A^	22.54 ± 0.06 ^B^	24.01 ± 0.06 ^a^	22.95 ± 0.06 ^b^
***Functional properties***				
Oil binding capacity (g oil/g sample)	2.65 ± 0.06 ^B^	3.25 ± 0.02 ^A^	2.76 ± 0.02 ^b^	3.32 ± 0.06 ^a^
Foaming ability (%)	4.7 ± 0.30 ^B^	13.4 ± 0.30 ^A^	4.7 ± 0.10 ^b^	11.7 ± 1.90 ^a^
Foaming stability (%)	55.6 ± 7.90 ^B^	100 ± 0.00 ^A^	33.3 ± 23.60 ^b^	61.1± 7.90 ^a^
Emulsifying ability (%)	96.7 ± 0.90 ^B^	100 ± 0.00 ^A^	94.7 ± 1.90 ^b^	100 ± 0.00 ^a^
Emulsifying stability (%)	69.0 ± 1.40 ^B^	75.6± 1.00 ^A^	68.4 ± 2.40 ^b^	76.6 ± 0.20 ^a^
***Antioxidant properties***				
DPPH radical-scavenging activity (% Increment, after in vitro digestion)	22.37 ± 0.01 ^B^	37.84 ± 0.01 ^A^	28.75 ± 0.01 ^b^	40.45 ± 0.01 ^a^
ABTS radical-scavenging activity(% Increment, after in vitro digestion)	12.75 ± 0.07 ^B^	37.86 ± 0.03 ^A^	3.94 ± 0.04 ^b^	28.46 ± 0.03 ^a^

Different letters in each row of each rice variety represent significantly different mean values (*p* < 0.05).

## Data Availability

The data presented in this study and supporting data are available on request from the corresponding author.
